# ddPCR provides a sensitive test compared with GeneXpert MTB/RIF and mNGS for suspected *Mycobacterium tuberculosis* infection

**DOI:** 10.3389/fcimb.2023.1216339

**Published:** 2023-12-01

**Authors:** Dan Zhang, Fei Yu, Dongsheng Han, Weizhen Chen, Lingjun Yuan, Mengxiao Xie, Jieyuan Zheng, Jingchao Wang, Bin Lou, Shufa Zheng, Yu Chen

**Affiliations:** ^1^ Department of Laboratory Medicine, the First Affiliated Hospital, Zhejiang University School of Medicine, Hangzhou, China; ^2^ Key Laboratory of Clinical In Vitro Diagnostic Techniques of Zhejiang Province, Hangzhou, China; ^3^ Institute of Laboratory Medicine, Zhejiang University, Hangzhou, China

**Keywords:** metagenomics next-generation sequencing, ddPCR, GeneXpert MTB/RIF, tuberculosis, latent TB infection

## Abstract

**Introduction:**

The Metagenomics next-generation sequencing (mNGS) and GeneXpert MTB/RIF assay (Xpert) exhibited a sensitivity for tuberculosis (TB) diagnostic performance. Research that directly compared the clinical performance of ddPCR analysis, mNGS, and Xpert in *mycobacterium tuberculosis* complex (MTB) infection has not been conducted.

**Methods:**

The study aimed to evaluate the diagnostic performance of ddPCR compared to mNGS and Xpert for the detection of MTB in multiple types of clinical samples. The final clinical diagnosis was used as the reference standard.

**Results:**

Out of 236 patients with suspected active TB infection, 217 underwent synchronous testing for tuberculosis using ddPCR, Xpert, and mNGS on direct clinical samples. During follow-up, 100 out of 217 participants were diagnosed with MTB infection. Compared to the clinical final diagnosis, ddPCR produced the highest sensitivity of 99% compared with mNGS (86%) and Xpert (64%) for all active MTB cases.

**Discussion:**

Twenty-two Xpert-negative samples were positive in mNGS tests, which confirmed the clinical diagnosis results from ddPCR and clinical manifestation, radiologic findings. Thirteen mNGS-negative samples were positive in ddPCR assays, which confirmed the clinical final diagnosis.ddPCR provides a higher sensitive compared to Xpert and mNGS for MTB diagnosis, as defined by the high concordance between ddPCR assay and clinical final diagnosis.

## Introduction

Tuberculosis (TB) is a communicable disease that is one of the major causes of death. Until the coronavirus disease 2019 (COVID-19) pandemic, TB was the leading cause of death from a single infectious agent, ranking above acquired immune deficiency syndrome (AIDS) (World Health Organization, 2021). *Mycobacterium tuberculosis* complex (MTB) infection is a serious health issue with high infectivity, morbidity, and mortality. The microbiological detection of MTB is critical to the definitive diagnosis of TB. Among the TB-diagnosed cases, only 57% of pulmonary TB cases are bacteriologically confirmed by sputum smear microscopy, culture, or rapid MTB DNA tests (World Health Organization, 2020). The TB cases without bacteriological confirmation may result in ineffective treatment. Traditional mycobacterial culture is time-consuming, and the acid-fast bacilli (AFB) smear has low sensitivity (Li et al., 2022). Nucleic acid amplification tests (NAATs) have been widely applied in the rapid diagnosis of MTB (Zhang et al., 2021). Xpert MTB/RIF (Cepheid, United States) based on RT-PCR assays and Truenat MTB (Molbio, India) assays are recommended by the World Health Organization (WHO) as a rapid molecular diagnostic test for the case of extrapulmonary and pulmonary infections (Penn-Nicholson et al., 2021; World Health Organization, 2021; Hong et al., 2022).

In recent years, the mNGS technique has been applied to detect causative pathogens without any prior suspicion of certain pathogens and to guide targeted antimicrobial therapy. It can be used in multiple clinical diagnoses, including tuberculosis (Shi et al., 2020; Li et al., 2022). In terms of MTB diagnostic performance, mNGS shows high efficiency as compared with Xpert in these cohort (Shi et al., 2020; Li et al., 2022). These studies showed that mNGS is better in cerebrospinal fluid (CSF) and bronchoalveolar lavage fluid (BALF) samples, only slightly worse in pleural effusion, pericardial effusion, and ascites (Zhou et al., 2019). Normally, for Xpert-negative patients, experienced clinicians could identify the MTB infection by analyzing the mNGS-positive results to see whether they were in accordance with the patient’s clinical manifestation and computed tomography (CT) images. For a high-priority pathogen like MTB, genus-specific read numbers ≥1 from mNGS-positive reporting threshold are normally considered to be credible reports. However, it was difficult to discriminate whether the false-positive results were caused by sampling contamination or by data analysis approaches (Miao et al., 2018). Additionally, mNGS has several drawbacks, including a long turnaround time, insufficient sensitivity in high background microorganisms, and cost-effectiveness considerations.

Compared with the conventional methods, the newly emerging droplet digital polymerase chain reaction (ddPCR) is observed with high sensitivity, precision, and accuracy for the identification of trace DNA in samples of low concentrations (Ushio et al., 2016; Li et al., 2020; Lyu et al., 2020). The ddPCR is proven to be fast enough to detect low-abundance MTB infection (Nyaruaba et al., 2019; Antonello et al., 2021). For suspected MTB infections without microbiological evidence, ddPCR is a good supplementary diagnosis for low-concentration MTB. Earlier studies have shown that mNGS and ddPCR demonstrate great promise in detecting MTB infections (Ushio et al., 2016; Nyaruaba et al., 2019; Cao et al., 2020; Yan et al., 2020; Liu et al., 2021; Zhu et al., 2021). However, the direct comparison of the ddPCR analysis with mNGS and Xpert in MTB infection is still scarce. The utility of ddPCR, mNGS, and Xpert for suspected clinical MTB infection has not been systematically evaluated.

In our study, a cohort of patients with suspected MTB infection was collected. The clinical application of ddPCR, mNGS, and Xpert methods for the accurate detection of MTB in the clinical samples was evaluated. The clinical diagnosis was used as the reference standard.

## Materials and methods

### Study participants

All patients with suspected of active MTB infections, including both pulmonary TB and extrapulmonary TB, were admitted to the First Affiliated Hospital, Zhejiang University School of Medicine from March 2021 to November 2021. Samples were collected from suspected infected sites of the enrolled patients. All clinical specimens were immediately sent for testing, including culture, Xpert, mNGS, ddPCR, and smear microscopy (for acid-fast bacilli). Fresh tissue, pleural effusion, pericardial effusion, CSF, ascites, and BALF samples were collected and divided into three aliquot parts for each sample.

The final clinical diagnosis of TB was made by infectious physicians or respiratory physicians based on the integration of the patient’s medical history, radiological findings, and laboratory findings (microbiological, molecular, and immunological tests) according to the China Clinical Treatment Guide for Tuberculosis (Association CM, 2005). There are two types of confirmed TB cases: microbiological test-confirmed TB cases and clinically diagnosed TB cases. For microbiologically confirmed TB cases, the patients were classified as microbiologically confirmed TB cases by the following results: MTB biopsy-positive and culture-positive, mNGS-positive, or Xpert-positive. For patients without microbiological evidence, the attending physician can only clinically diagnose the active MTB infection by combining the patient’s imaging findings, clinical manifestations, and other laboratory-related tests and exclude it from other diseases. The patient’s TB was finally confirmed by its responsiveness to anti-TB treatment at 1-month follow-up. The non-TB group included infections other than TB, non-infectious inflammatory diseases, tumors, and miscellaneous causes.

This study conformed to the ethical guidelines of the 1975 Declaration of Helsinki and was approved by the ethics committee of the First Affiliated Hospital, Zhejiang University School of Medicine.

### GeneXpert MTB/RIF detection

BALF samples were ground into a homogenate and liquefied. After centrifugating each BALF, pleural effusion, pericardial effusion, and ascites were collected as 2-mL sedimentation in DNase-free tubes. Tissue was shredded using a blade and by grinding. The Xpert MTB/RIF assay was performed on the GeneXpert system (Cepheid, Sunnyvale, CA, USA) following the manufacturer’s instructions. The results were determined by the fluorescence signal measured using the GeneXpert system and the built-in algorithm.

### Sample processing and droplet digital PCR

Briefly, the same pre-processing was conducted with BALF and tissue, pleural effusion, pericardial effusion, and ascites. Pleural effusion, pericardial effusion, and ascites were collected as 2-mL sedimentation in DNase-free tubes. Bead-beating for cell lysis was required for all samples and using the host depletion approach before nucleic acid extraction. Following are additional details of bead-beating lysis: bead size: 1 mm and 3 mm zirconium beads; time setting: running time = 70 s, pause time = 30 s; number of cycles = 8; TGrinder H24 Tissue Homogenizer (Tiangen). After the bead-beating process, DNA was extracted using TIANamp Micro DNA Kit (DP316, Tiangen Biotech, Beijing, China) according to the manufacturer’s protocol. DNA was eluted in 50 μL of elution buffer and used for the ddPCR assay and mNGS analysis promptly on the same day. The ddPCR analysis was performed using Targeting One Droplet Digital PCR System according to the manufacturer’s protocol. Briefly, a 30-μL reaction mixture and 180 μL oil were loaded onto the droplet. The mixture was prepared for the emulsion generation using the TargetingOne Drop Maker M1 (TargetingOne, Beijing, China), and approximately 40,000 droplets were obtained. After 10 min at 95°C, a total of 40 cycles of the PCR reaction were carried out in a Bio-Rad PTC 200 thermal cycler according to the following procedure: 94°C for 30 s, 57°C for 1 min, and 12°C cooling for 5 min. The PCR droplets were then loaded onto Chip Reader R1 (TargetingOne, Beijing, China) to analyze the FAM (488-nm laser) fluorescence intensity for IS1081 and VIC (532-nm laser) for IS6110 fluorescence intensity for each droplet. The cluster plots were calculated using TargetingOne analysis software (TargetingOne, Beijing, China). Each sample was performed in duplicate. The threshold levels for selecting positive droplets were determined by the fluorescence intensities of the standard droplets. At least one copy of positive droplet MTB gene IS6110 or IS1081 was required for a positive test result. The threshold levels for selecting positive droplets were determined by a density-watershed algorithm method, which was developed for the accurate, automatic, and un-supervised classification of two/four-ddPCR data (Zhu et al., 2019).

### Accuracy and limit of detection of droplet digital PCR assay

To verify the performance of MTB detection and quantification, a national reference for the PCR-based detection of *Mycobacterium tuberculosis* was purchased from the National Institutes for Food and Drug Control (Beijing, China; lot 230030-202004). The limits of detection (LODs) for the ddPCR assays were measured using four serial dilutions (1, 10, 10^2^, and 10^3^ copies/mL) of the MTB around the LOD. The LODs were calculated by using probit analysis for 95% positive result. The coefficient of determination of the MTB quantification was assessed for both IS6110 and IS1081 by using linear regression analysis. To determine the specificity and cross-reactivity of the MTB ddPCR assays, the DNA of cultured nontuberculous mycobacteria strains (NTMs), other bacterial strains, and negative controls, respectively, were also added in each run. The negative controls belonged to samples collected from individuals with no clinical and bacteriological signs of MTB infection.

### Metagenomic next-generation sequencing and analysis

The synthesized DNA fragment was used as an internal control, and DNase-free water and samples from three healthy subjects were spiked with the internal control to monitor for external or reagent microbial contamination and cross-sample contamination. The same DNA was quantified using Qubit dsDNA HS Assay Kit (Life Technologies, CA, USA). DNA libraries were constructed by using Nextera XT Library Preparation Kit (Illumina, CA, USA) following the manufacturer’s protocol. Sequencing was performed on an Illumina Nextseq CN550 platform (Illumina, CA, USA), resulting in 15–20 million reads (75 bp) for each sample. The sequence data was analyzed by using Weiyuan-MG v1.0 mNGS software (Weiyuan, Weiyuan Biotechnology, Guangzhou, China) which contains a proprietary curated database consisting of more than 20,000 reference microbial genomes.

### Criteria for a positive mNGS result

The sequencing results of each sample are categorized into four tables, with each table representing bacteria, fungi, viruses, and parasites. The mNGS identified a microbe at the species level whose read count was among the top 10 in the complete list of species as well as a microbe at the species level whose coverage rate was 10 times greater than that of any other microbe. We identified the causative pathogen according to the pipeline described previously (Miao et al., 2018). MTB was considered positive when at least one read was mapped to either the species or genus level due to the difficulty of DNA extraction and the low possibility for contamination.

### Statistical analysis

SPSS statistical package 22.0 software was used for database management and statistical analyses. Continuous variables were described by means when they conform to the *t*-test. The *t*-test was used to analyze normally distributed continuous variables, whereas the Mann–Whitney *U*-test was used to analyze nonnormally distributed continuous variables. Categorical variables were reported as frequencies and percentages and analyzed using chi-square test. The sensitivity and specificity of the different methods were assessed. The results were presented with a range of 95% confidence intervals. Analysis of variance test was applied to compare differences across subgroups. A *P*-value less than 0.05 was considered statistically significant.

## Results

### Patients’ characteristics

**Figure 1:**
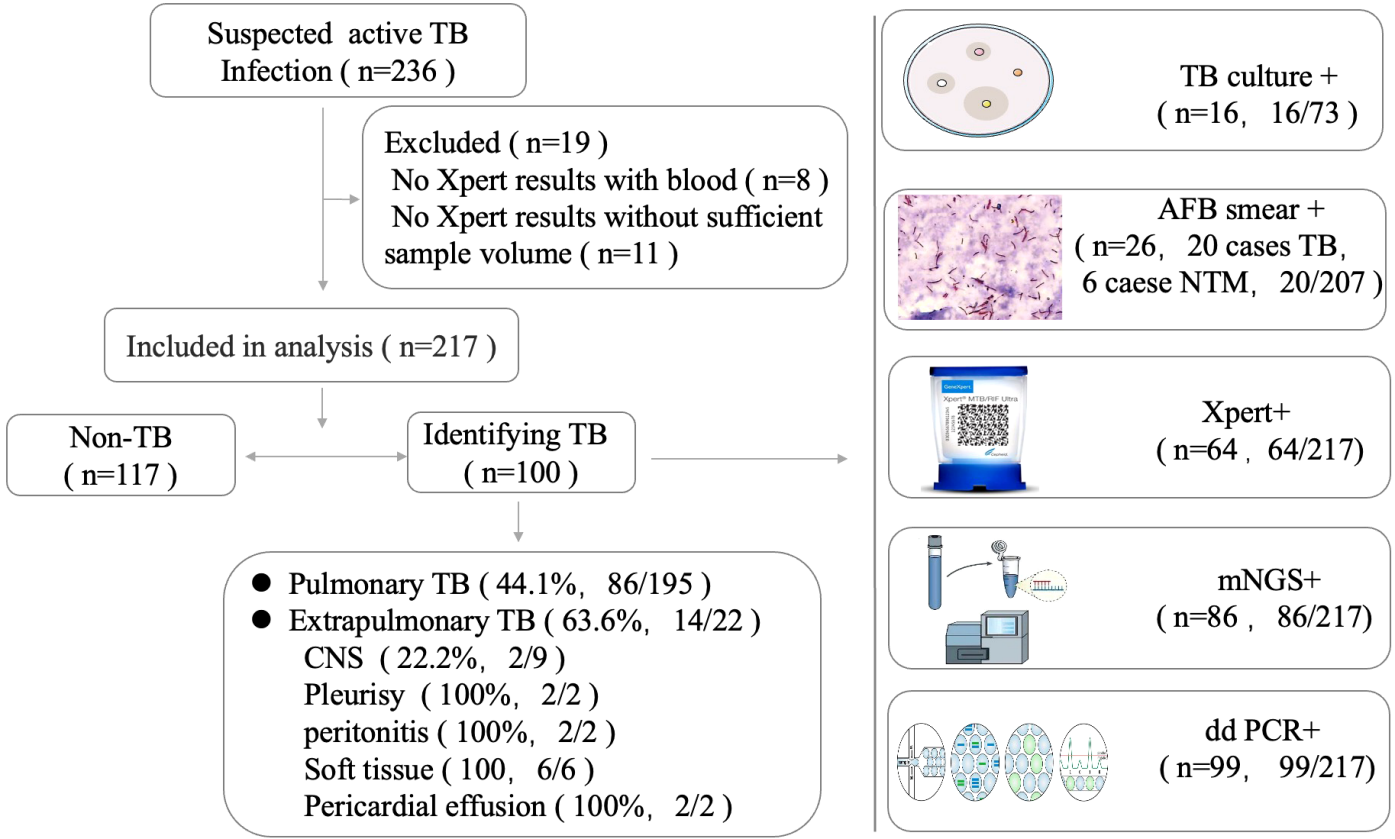
Study design.

The demographic and clinical characteristics of the population included in the study are presented in **Table 1**. We enrolled 236 patients suspected to have active MTB infections. In total, 19 patients were excluded due to the limited specimen that was not enough for all three tests and the priority test that was chosen by their attending physician (**Figure 1**). Samples were retrieved mainly among male patients (68.2%) with a median age of 56.3 years (interquartile range: 50–68). A total of 217 patients initially suspected of having active pulmonary MTB infection underwent bronchoscopy during hospitalization, and 195 clinical lavage fluid samples and 22 other specimens (pleural effusion, pericardial effusion, CSF, ascites, and tissue) were obtained for the tests. All the clinical samples were subjected to tuberculous diagnosis using ddPCR, Xpert, mNGS, and traditional diagnostic methods (**Figure 1**). Moreover, 100 of 217 participants had a final clinical diagnosis of active pulmonary MTB infection.

**Table 1:** Baseline characteristics of the population.

	TB (n=100)	Non-TB (n=117)
**Age (mean), years**	54.6	57.9
**Sex-male, n (%)**	77 (77%)	71 (60.7%)
T. SPOT TB (+), n (%)	69 (69%)	97 (82.90)
Pulmonary samples, n		
BALF	84	111
Lung biopsy tissue	6	0
Extrapulmonary samples, n		
CSF	2	7
Pleural fluid	2	0
Ascites	2	0
pericardial effusion	2	0
AFB (+)	26	6
**Mycobacterial culture, n**	22	6

### Performance of droplet digital PCR-based assays

#### Assay linearity and limit of detection

**Figure 2:**
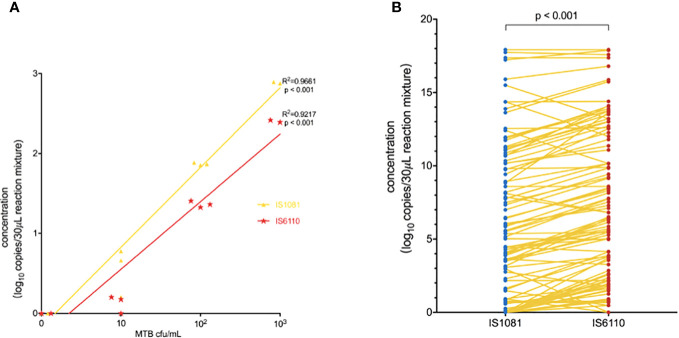
**(A)** Linear correlation of the observed IS1081 and IS6110 load, expressed as (log _10_ copies/30 μL reaction mixture) concentration. **(B)** Duplex amplification of MTB IS6110 and IS1081 DNA in clinical samples by ddPCR. The differences in copy number of IS6110-dPCR and IS1081-dPCR were analyzed by using the Wilcoxon test.

The linearity of the IS6110 and IS1081 duplex ddPCR assays was tested by quantifying serial dilutions of a known amount of MTB DNA. The probit analysis of 95% positivity in 10 replicates in three different runs at five concentrations showed that the MTB ddPCR assays showed an LOD of three copies per reaction. Both IS6110 (*R* ^2^ = 0.9661, *p* < 0.001) and IS1081 (*R* ^2^ = 0.9217, *p* < 0.001) targeted tests were correlated with MTB DNA quantification (**Figures 2A, B**). No signal was detected in any of the 15 certainly negative samples nor in the added nontuberculous extract for both IS6110 and IS1081 targets. A representative example of Quanta soft panel for IS6110 and IS1081 was obtained by the positive MTB DNA control, and four positive samples were displayed. The specificities of IS6110 and IS1081 ddPCR for the MTB for the NTMs and other bacterial strains were confirmed (**Table 2**).

**Table 2:** Amplification of NTMs and other bacterial strains in ddPCR.

Strain	Amplification Target	
	IS6110	IS1081
M. avium	N	N
M. kansasii	N	N
M.terraccomplex	N	N
M. shimoidei	N	N
M. asiaticum	N	N
M. scrofulaceum	N	N
M.gordonae	N	N
M.chelonei	N	N
M. Fortuitum	N	N
M. phlei	N	N
Nocardia brasiliensis	N	N
Corynebacterium pekinense	N	N
Streptococcus pneumoniae	N	N

NTM, nontuberculous mycobacteria.

N, negative.

#### Results of ddPCR in the detection of MTB DNA for clinical samples

**Figure 3:**
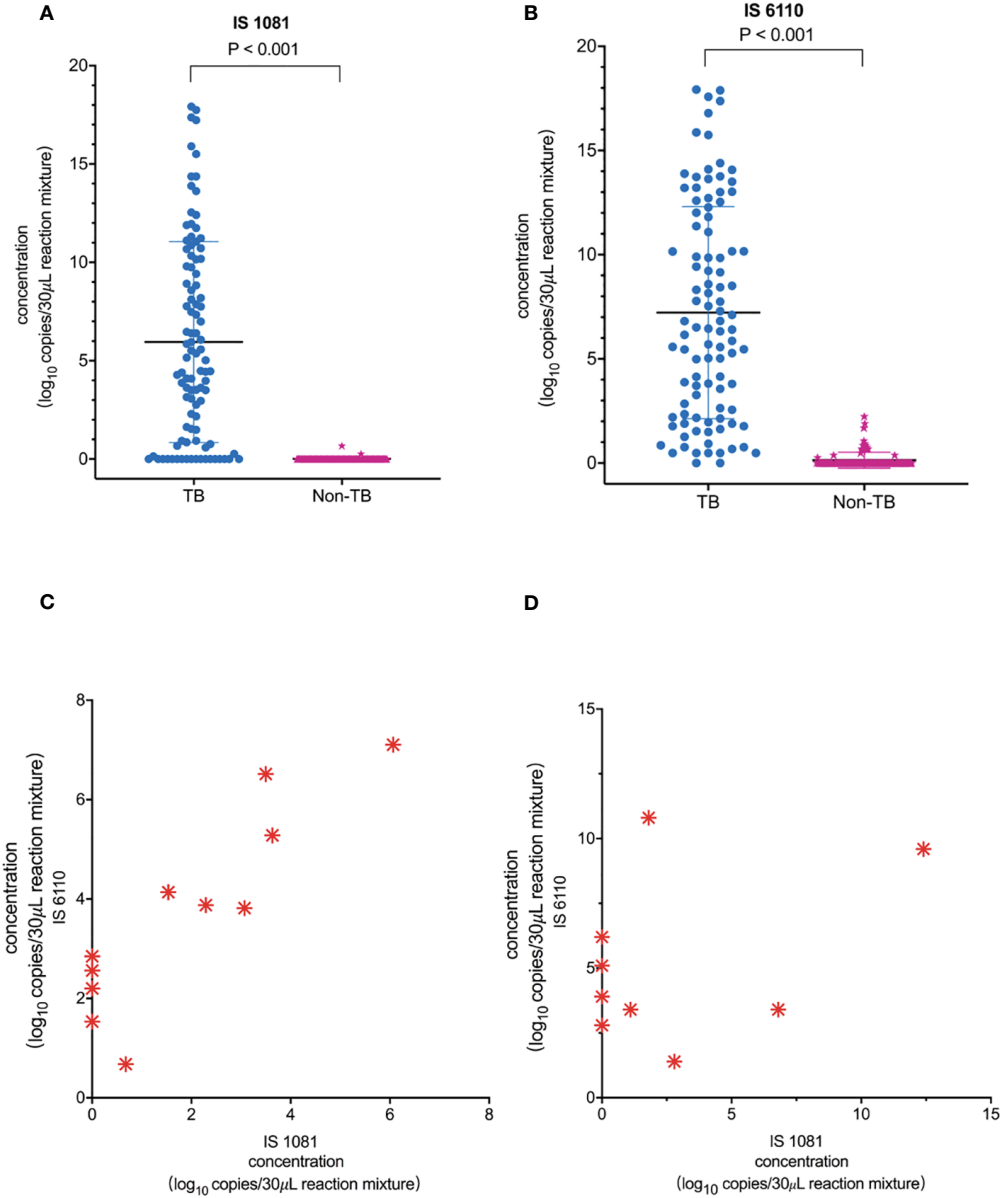
Quantification of MTB IS1081 and IS6110 DNA in clinical samples by ddPCR. **(A)** IS1081 and **(B)** IS6110 copy number in the clinical samples of total TB patients and non-TB patients, respectively (Mann–Whitney *U*-test). The results were considered significant when the *p*-value was 0.05 or lower. **(C)** All one-read MTB samples were detected by mNGS as positive and confirmed the final clinical diagnosis of TB. Only five of 11 one-read MTB samples were detected to be positive by Xpert. **(D)** Nine clinical samples were detected to be positive by ddPCR and confirmed the final clinical diagnosis of TB.

As shown in **Figures 3A, B**, the number of copies detected in the TB group was significantly higher than that in the non-TB group: median (minimum, maximum) for IS6110, 79.7 (0.0, 248522.0) copies/30 μL of reaction mixture in the TB group vs. 0.0 (0.0, 4.7) copies/30 μL of reaction mixture in the non-TB group, *p* < 0.001. Similar results were also observed for the IS1081 assay: 22.4 (0.0, 248522.0) copies/30 μL of reaction mixture in the TB group vs. 0.0 (0.0, 1.6) copies/30 μL of reaction mixture in the non-TB group, *p* < 0.001 (**Figures 3A, B**). Our results also showed that the sensitivity of the IS6110-ddPCR assay for total TB was higher than that of the IS1081-ddPCR assay in the low loads MTB, especially in samples with only one read of detected MTB in the mNGS-positive samples and only in the ddPCR-positive samples (**Figures 3C, D**).

#### Diagnosis performance of ddPCR-based assays against Xpert and mNGS

**Figure 4:**
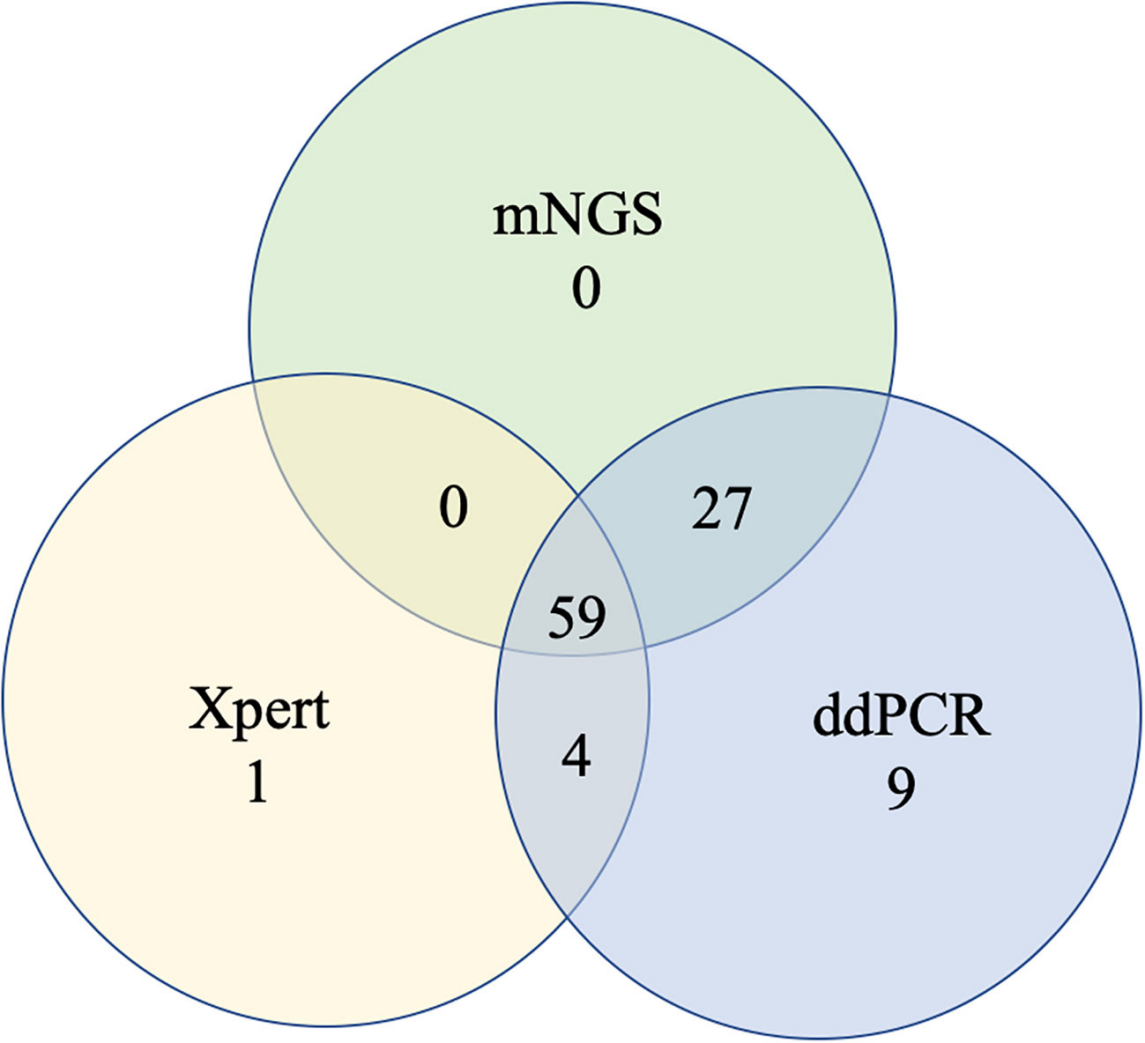
Venn diagram of overlap in TB diagnostics.

To further measure the sensitivity and specificity of ddPCR-based assays against Xpert and mNGS, we assessed the diagnostic performance for ddPCR, Xpert, mNGS, and traditional methods and compared them with the final clinical diagnosis (**Table 3**; **Figure 4**). In total, 100 of 217 participants had a final clinical diagnosis of active pulmonary MTB infection. ddPCR produced the highest sensitivity of 99% among the three nucleic acid amplification tests, followed by mNGS (86%) and Xpert (64%), yet for the specificity results of the tests, mNGS had the highest specificity of 100%. With only four false-positive results, ddPCR had a specificity of 96.6% in our study. MTB was detected by ddPCR in 9.0% (9/100) Xpert- and mNGS-negative samples. Only three BAL and one ascites sample with Xpert-positive results were negative in the mNGS tests. A total of 22 Xpert-negative samples were positive in mNGS tests, which further confirmed the final clinical diagnosis results by ddPCR, clinical manifestation, and radiologic findings. Furthermore, 13 mNGS-negative samples were positive in ddPCR assays, which confirmed the final clinical diagnosis. The negative predictive value of ddPCR, mNGS, and Xpert for the detection of MTB was 99.1% (95%CI: 94.5–100%), 89.3% (95%CI: 82.4–93.8%), and 76.4% (95%CI: 68.8–82.8%), respectively. It also showed a higher sensitivity of ddPCR. Six cases of NTMs were simultaneously detected as negative both in mNGS and ddPCR. The diagnosis results were confirmed by using AFB smear and culture.

**Table 3:** Diagnostic performance of MTB for mNGS, Xpert, ddPCR and traditional methods compared with the clinical final diagnosis.

Methods	samlpe size (n)	the final clinical diagnosis for TB		Statistical analysis			
		Yes, (n=100)	No, (n=117)	Sensitivity, % (95% CI)	Specificity, % (95% CI)	PPV, % (95% CI)	NPV, % (95% CI)
mNGS (Positive)	217	86	0	86 (77.2-91.8)	100 (96.0-100)	100 (94.6-100)	89.3 (82.4-93.8)
(Negative )		14	117				
Xpert (Positive)	217	64	0	64 (53.7-73.1)	100 (96.0-100)	100 (92.9-100)	76.4 (68.8-82.8)
(Negative )		36	117				
ddPCR (Positive)	217	99	4	99 (93.8-99.9)	96.6 (91.0-98.9)	100 (89.8-98.7)	99.1 (94.5-100)
(Negative )		1	113				
AFB smear (Positive)	207	20	0	22.4(14.6-32.8)	100 (95.6-100)	100 (80.0-100)	61.8(54.3-68.9)
(Negative )		74	117				
Culture (Positive)	73	16	0	43.2(27.5-60.4)	100 (88.0-100)	100 (75.9-100)	63.2 (49.3-75.2)
(Negative )		84	117				

NPV, negative predictive value; PPV, positive predictive value.

## Discussion

The current molecular diagnostic techniques for TB, including PCR and mNGS, have insufficient sensitivity to detect samples with low bacterial loads and showed limited efficacy, although the mNGS and Xpert assays exhibited a sensitivity for TB diagnostic performance. Some suspected tuberculosis diagnoses still lacked etiological evidence. ddPCR is an excellent nucleic acid quantification technique with high sensitivity (Zhao et al., 2022). However, directly evaluating the diagnostic performance of ddPCR with mNGS and Xpert in pulmonary MTB infection is still rare so far. To address this issue, we conducted a single-center study to analyze the performance of ddPCR for suspected clinical specimens of MTB infection and compared it with mNGS, Xpert, and the final clinical diagnosis. Our results proved that the performance of ddPCR-assays is a rapid and sensitive molecular test for the detection of low-abundance MTB infection. Among these NAAT methods, the ddPCR results are in best accordance with the final clinical diagnosis of MTB infection, which could potentially improve the consistency of clinical diagnosis.

Although previous studies have shown the potential of the application of mNGS in identifying MTB infections (Zhou et al., 2019; Shi et al., 2020; Liu et al., 2021; Zhu et al., 2021), no consensus on the detection capacity of mNGS for MTB has been reached. For high-priority pathogens, MTB was considered positive when at least one read was assigned to the species or genus level due to the difficulty of DNA extraction and the low possibility of contamination (Miao et al., 2018; Shi et al., 2020). In our study, it was difficult for 11 clinical samples to detect one-read MTB by mNGS and identify MTB infections. Experienced clinics also analyzed the mNGS results to see whether it was in accordance with the imaging findings, clinical manifestation, and diagnosis. MTB specimens with only one read were detected not only with low loads of IS6110 and IS1081 by ddPCR but also in six Xpert-negative specimens. It demonstrated that mNGS had an improved diagnostic performance in suspected MTB. mNGS was useful for the distinction between MTB and NTM.

In terms of TB diagnostic performance, mNGS is inferior to Xpert in our overall cohort, which is better in BAL. The sensitivity of ddPCR and mNGS using the same nucleic acid was higher than that of mycobacterial Xpert. Part of the reason may be in the sample DNA extraction with bead-beating processing. Previous studies have shown that the detection rate can be greatly improved in laboratories by adding a bead-beating process (Charalampous et al., 2019). Mechanical wall breaking can increase the yield of microbes at low concentrations, especially for MTB with a hard cell wall. However, some studies showed that mNGS is not superior to Xpert in detecting MTB (Shi et al., 2020; Liu et al., 2021). In our study, the sensitivity of mNGS (86%) was higher than that of Xpert (64%). mNGS is superior to Xpert in detecting MTB, which could be caused by adding a host DNA depletion process. The results indicated that adding a host DNA depletion process in an mNGS protocol might reduce the risk of false-negatives in testing low-microbial-biomass samples (Han et al., 2021) because different levels of background commensal/human DNA could potentially result in different LODs.

For suspected TB, some clinicians prescribe early empirical treatment based on the appearance of the patient’s imaging findings and clinical manifestations which cannot replace an etiological confirmation. It is a challenge to detect low-abundance MTB samples for mNGS or Xpert. Therefore, the results of ddPCR were useful in providing appropriate treatment to patients in the early stage or latent TB infection (Song et al., 2018), supporting the clinical decision of treatment. In our study, ddPCR showed superior sensitivity in detecting pathogens compared with traditional diagnosis methods. Of the 14 false-negative findings with mNGS, ddPCR showed a higher sensitivity in probable MTB. For ddPCR specificity, 22 cases of NTM were detected by mNGS-positive and clinically diagnosed NTM cases, but only six cases were confirmed by culture and AFB smear. There were two false-positive ddPCR in the clinically diagnosed NTM cases. Only four IS6110 and IS1081 ddPCR-positive samples and were finally diagnosed with MTB infection after tracking the cases for half a year. These showed a higher sensitivity in probable and possible MTB. In addition, another four IS6110 and IS1081 ddPCR-positive samples which cannot be excluded as MTB were finally diagnosed after tracking the cases for half a year. It was demonstrated that ddPCR has a higher sensitivity.

In the IS6110 and IS1081 duplex ddPCR systems, IS6110 accounts for many more copies in the genome of clinical MTB samples than IS1081. It has been reported that the testing of IS6110 was not promising in clinical MTB samples from several areas such as Southeast Asia because some MTB strains only had one copy of IS6110 in their genome (Lyu et al., 2020). Some clinically isolated strains were found to have no IS6110 element, which accounts for approximately 5% of the total isolates (Huyen et al., 2013). Several studies have indicated MTB strains that typically contain multiple copies of IS6110 (up to 25 per genome), although strains with only a single copy or no copies have also been identified (Alonso et al., 2013). It can explain our results observed, such that IS6110-PCR has a higher sensitivity than IS1081 qPCR. It will need more advanced research in the future. Therefore, the sensitivity of joint detection was improved with the IS6110 and IS1081 assay for MTB with low loads. The disadvantages of ddPCR over other molecular methods need to be mentioned (1): higher chances of sample contamination and (2) ddPCR implementation needs specialized personnel.

Despite these promising results, our study has some limitations. First, our study had a relatively small extrapulmonary sample size; therefore, more future studies are required to understand the diagnostic value of mNGS in extrapulmonary TB. Due to interference, Xpert cannot detect tuberculosis in plasma, while mNGS and ddPCR were used for detecting TB and confirmed the final clinical diagnosis of MTB in four cases using plasma samples. Thus, plasma was not included in the study. Second, antibiotic resistance genes were not analyzed by mNGS and cannot help more targeted treatment for pulmonary TB. Third, the single-center study had limitations. Multi-center studies would be needed to explore universal criteria or guidelines of mNGS in active TB infections in the future. Finally, despite the low sensitivity of conventional methods, the study has not systematically included conventional methods such as culture and AFB. However, mNGS and Xpert showed an overall superior advantage over conventional methods and significantly improved the etiology diagnosis of both MTB.

In summary, our data show that the mNGS of the clinical sample represents a potential step forward in the MTB diagnosis of suspected pulmonary TB and extrapulmonary TB. ddPCR has been sensitive and has excellent accuracy for the rapid diagnosis of MTB in detecting low-pathogen-load samples. Among these methods, ddPCR is strongly recommended in clinical practice for the diagnosis of TB and the follow−up of positive patients.

## Data availability statement

The original contributions presented in the study are included in the article/supplementary materials, further inquiries can be directed to the corresponding author/s.

## Ethics statement

The study was conducted in accordance with the Declaration of Helsinki and was approved by FAHZU institutional review board (IIT20220714A).

## Author contributions

YC and SZ conceived and designed the study. BL and DZ obtained clinical samples and defined the clinical labels. FY and DH performed statistical analysis. WC and DZ wrote the manuscript. LY, MX, JZ and JZ provided clinical expertise and guidance on the study design. YC supervised the project. All authors have accepted responsibility for the entire content of this manuscript and approved its submission.

## References

[B1] Alonso H. Samper S. Martín C. Otal I. (2013). Mapping IS6110 in high-copy number Mycobacterium tuberculosis strains shows specific insertion points in the Beijing genotype. BMC Genomics 14, 422. doi: 10.1186/1471-2164-14-422 23800083 PMC3701491

[B2] Antonello M. Scutari R. Lauricella C. Renica S. Motta V. Torri S.. (2021). Rapid detection and quantification of *Mycobacterium tuberculosis* DNA in paraffinized samples by droplet digital pcr: a preliminary study. Front. Microbiol. 12, 727774. doi: 10.3389/fmicb.2021.727774 34589075 PMC8475183

[B3] Association CM. (2005). China clinical treatment guide for tuberculosis. Beijing, China: People’s Medical Publishing House.

[B4] Cao Z. Wu W. Wei H. Gao C. Zhang L. Wu C.. (2020). Using droplet digital PCR in the detection of Mycobacterium tuberculosis DNA in FFPE samples. Int. J. Infect. Dis. 99, 77–83. doi: 10.1016/j.ijid.2020.07.045 32738487

[B5] Charalampous T. Kay G. Richardson H. Aydin A. Baldan R. Jeanes C.. (2019). Nanopore metagenomics enables rapid clinical diagnosis of bacterial lower respiratory infection. Nat. Biotechnol. 37 (7), 783–792. doi: 10.1038/s41587-019-0156-5 31235920

[B6] Han D. Diao Z. Lai H. Han Y. Xie J. Zhang R.. (2021). Multilaboratory assessment of metagenomic next-generation sequencing for unbiased microbe detection. J. Adv. Res. 38, 213–222. doi: 10.1016/j.jare.2021.09.011 35572414 PMC9091723

[B7] Hong J. Lee H. Menon N. Lim C. Lee L. Ong C. (2022). Point-of-care diagnostic tests for tuberculosis disease. Sci. Transl. Med. 14 (639), eabj4124. doi: 10.1126/scitranslmed.abj4124 35385338

[B8] Huyen M. Tiemersma E. Kremer K. de Haas P. Lan N. Buu T.. (2013). Characterisation of Mycobacterium tuberculosis isolates lacking IS6110 in Viet Nam. Int. Tuberc Lung Disease. 17 (11), 1479–1485. doi: 10.5588/ijtld.13.0149 24125454

[B9] Li Y. Jiao M. Liu Y. Ren Z. Li A. (2022). Mycobacterium tuberculosisApplication of metagenomic next-generation sequencing in *Mycobacterium tuberculosis* infection. Front. Med. 9, 802719. doi: 10.3389/fmed.2022.802719 PMC9010669 35433724

[B10] Li Z. Pan L. Lyu L. Li J. Jia H. Du B.. (2020). Diagnostic accuracy of droplet digital PCR analysis of cerebrospinal fluid for tuberculous meningitis in adult patients. Clin. Microbiol. Infect. 26 (2), 213–219. doi: 10.1016/j.cmi.2019.07.015 31336201

[B11] Liu X. Chen Y. Ouyang H. Liu J. Luo X. Huang Y.. (2021). Tuberculosis Diagnosis by Metagenomic Next-generation Sequencing on Bronchoalveolar Lavage Fluid: a cross-sectional analysis. Int. J. Infect. Dis. 104, 50–57. doi: 10.1016/j.ijid.2020.12.063 33359946

[B12] Lyu L. Li Z. Pan L. Jia H. Sun Q. Liu Q.. (2020). Evaluation of digital PCR assay in detection of M.tuberculosis IS6110 and IS1081 in tuberculosis patients plasma. BMC Infect. Dis. 20 (1), 657. doi: 10.1186/s12879-020-05375-y 32894079 PMC7487892

[B13] Miao Q. Ma Y. Wang Q. Pan J. Zhang Y. Jin W.. (2018). Microbiological diagnostic performance of metagenomic next-generation sequencing when applied to clinical practice. Clin. Infect. Dis. 67 (suppl_2), S231–S240. doi: 10.1093/cid/ciy693 30423048

[B14] Nyaruaba R. Mwaliko C. Kering K. K. Wei H. (2019). Droplet digital PCR applications in the tuberculosis world. Tuberculosis (Edinb). 117, 85–92. doi: 10.1016/j.tube.2019.07.001 31378274

[B15] Penn-Nicholson A. Gomathi S. Ugarte-Gil C. Meaza A. Lavu E. Patel P.. (2021). A prospective multicentre diagnostic accuracy study for the Truenat tuberculosis assays. Eur. Respir. J. 58, 2100526. doi: 10.1183/13993003.00526-2021 34049948 PMC8607906

[B16] Shi C. L. Han P. Tang P. J. Chen M. M. Ye Z. J. Wu M. Y.. (2020). Clinical metagenomic sequencing for diagnosis of pulmonary tuberculosis. J. Infect. 81 (4), 567–574. doi: 10.1016/j.jinf.2020.08.004 32768450

[B17] Song N. Tan Y. Zhang L. Luo W. Guan Q. Yan M.. (2018). Detection of circulating Mycobacterium tuberculosis-specific DNA by droplet digital PCR for vaccine evaluation in challenged monkeys and TB diagnosis. Emerging Microbes infections. 7 (1), 78. doi: 10.1038/s41426-018-0076-3 29691363 PMC5915492

[B18] Ushio R. Yamamoto M. Nakashima K. Watanabe H. Nagai K. Shibata Y.. (2016). Digital PCR assay detection of circulating Mycobacterium tuberculosis DNA in pulmonary tuberculosis patient plasma. Tuberculosis (Edinb) 99, 47–53. doi: 10.1016/j.tube.2016.04.004 27450004

[B19] World Health Organization. (2020). Global tuberculosis report. Geneva, Switzerland: World Health Organization.

[B20] World Health Organization. (2021). Global tuberculosis report. Geneva, Switzerland: World Health Organization.

[B21] Yan L. Sun W. Lu Z. Fan L. (2020). Metagenomic Next-Generation Sequencing (mNGS) in cerebrospinal fluid for rapid diagnosis of Tuberculosis meningitis in HIV-negative population. Int. J. Infect. Dis. 96, 270–275. doi: 10.1016/j.ijid.2020.04.048 32339718

[B22] Zhang Z. Du J. Liu T. Wang F. Jia J. Dong L.. (2021). EasyNAT MTC assay: A simple, rapid, and low-cost cross-priming amplification method for the detection of mycobacterium tuberculosis suitable for point-of-care testing. Emerg. Microbes infections. 10 (1), 1530–1535. doi: 10.1080/22221751.2021.1959271 PMC8330774 34288833

[B23] Zhao Z. Wu T. Wang M. Chen X. Liu T. Si Y.. (2022). A new droplet digital PCR assay: improving detection of paucibacillary smear-negative pulmonary tuberculosis. Int. J. Infect. Dis. 122, 820–828. doi: 10.1016/j.ijid.2022.07.041 35870796

[B24] Zhou X. Wu H. Ruan Q. Jiang N. Chen X. Shen Y.. (2019). Clinical Evaluation of Diagnosis Efficacy of Active Mycobacterium tuberculosis Complex Infection via Metagenomic Next-Generation Sequencing of Direct Clinical Samples. Front. Cell Infect. Microbiol. 9. doi: 10.3389/fcimb.2019.00351 PMC6813183 31681628

[B25] Zhu N. Zhou D. Li S. (2021). Diagnostic accuracy of metagenomic next-generation sequencing in sputum-scarce or smear-negative cases with suspected pulmonary tuberculosis. BioMed. Res. Int. 2021, 9970817. doi: 10.1155/2021/9970817 34527747 PMC8437628

[B26] Zhu X. Su S. Fu M. Peng Z. Wang D. Rui X.. (2019). A density-watershed algorithm (DWA) method for robust, accurate and automatic classification of dual-fluorescence and four-cluster droplet digital PCR data. Analyst. 144 (16), 4757–4771. doi: 10.1039/c9an00637k 31290860

